# Safety and Efficacy of GLP-1 Agonists in a Cohort of Patients with Fontan Circulation

**DOI:** 10.1007/s00246-024-03700-9

**Published:** 2024-11-12

**Authors:** Andrew M. Freddo, Humera Ahmed, Jonathan B. Edelson, Juan M. Ortega-Legaspi, Sumeet Vaikunth

**Affiliations:** 1 Department of Cardiology, Children’s Hospital of Philadelphia, Philadelphia, PA USA; 2 Division of Cardiovascular Medicine, Hospital of the University of Pennsylvania, Philadelphia, PA USA

**Keywords:** Adult congenital heart disease, Fontan, GLP-1 agonists, Fontan failure, Obesity

## Abstract

There are a growing number of adult patients palliated to a Fontan circulation. As these patients age, many develop symptomatic heart failure (d’Udekem et al in Circulation 130:S32–S38, 2014) that is exacerbated by acquired comorbidities such as obesity and hypertension. Increased body mass index (BMI) and adiposity have been associated with worse hemodynamics and clinical outcomes in these patients (Yogeswaran et al in J Am Hear Assoc: Cardiovasc Cerebrovasc Dis 12:e026732, 2023). Recently, glucagon-like peptide-1 (GLP-1) agonists, originally developed to treat diabetes mellitus, received FDA approval for weight loss and have been shown to reduce the risk of cardiovascular events in the general population (Vilsbøll et al in BMJ 344:d7771, 2012). There are limited data on these medications in patients with a Fontan circulation. We conducted a retrospective review of adults with Fontan circulation followed in our adult congenital heart disease (ACHD) clinic between 2009 and 2023 and identified 8 patients prescribed GLP-1 agonists. We found that GLP-1 agonists were well-tolerated and led to modest reduction in weight and blood pressure. Further study of the use of these medications in this population is warranted.

## Cases

There were eight patients with Fontan circulation who were prescribed GLP-1 agonists. Clinical data are summarized in the Table 1. Five patients (62.5%) were female, and age at initiation ranged from 20 to 39 years old (median 32). Most patients had a systemic right ventricle (6/8, 75%). Starting BMI ranged from 24.9 to 52.5 kg/m^2^ (median 37.2). Baseline oxygen saturation at treatment initiation ranged from 87 to 94% (median 91%). Comorbidities included obesity (7, 87.5%), cirrhosis (4, 50%), anxiety and/or depression (4, 50%), and diabetes mellitus or pre-diabetes (3, 37.5%). Patients most commonly were also taking aspirin (6, 75%) and angiotensin converting enzyme inhibitors (6, 75%). Three patients (37.5%) were taking a loop diuretic and three (37.5%) were taking a beta blocker. Almost all patients on a GLP-1 agonist were prescribed semaglutide (7, 87.5%). Four patients’ (50%) primary care physicians, 3 patients’ (37.5%) endocrinologists, and 1 patient’s (12.5%) cardiologist prescribed the GLP-1 agonist. Nearly all patients (7, 87.5%) were started on a GLP-1 agonist for weight loss, with the remaining patient taking it for diabetes. Time of use ranged from 117 to 1181 days (median 265.5).

**Table 1:** Summary of patients in our series, including changes in their weight and blood pressure as well as underlying cardiac diagnosis, key morbidities, and other medications

Patient	Age/sex	Diagnosis	Key comorbidities	Other medications	GLP1 used	Year treatment started	Treatment time (days)	Initial BMI; weight change (kg, %)	SBP change (mmHg)	Side effects
Median [IQR]	32 [30.5–35.5]						265.5 [194.5–349.25]	37.2 [34.25 to 41.05]	− 9.5 [− 0.25 to − 16.5]	
1	29/F	HLHS	Hypertension, Cirrhosis, Obesity	Aspirin, Spironolactone, Enalapril	Semaglutide	2023	287	35.5 + 4.5 kg, + 4.8%	− 18	None reported
2	33/F	PA/IVS	Hyperlipidemia, MI, PH, OSA, Cirrhosis, Pre-diabetes	Aspirin, Lisinopril, Tadalafil, Torsemide	Semaglutide	2023	380, ongoing	35.3 − 5.6 kg, − 5.6%	− 16	GI discomfort
3	31/M	DORV/HLHS	Diabetes	Aspirin, Lisinopril, Metformin	Semaglutide	2024	117, ongoing	40.2 + 1.8 kg, + 1.5%	− 2	None reported
4	39/F	DORV	Anxiety, Diabetes, Thrombus	Digoxin, Diltiazem, Enalapril, Insulin, Metoprolol, Nadolol, Warfarin	Dulaglutide	2021	1181, ongoing	24.9 − 1.4 kg, − 2.1%	− 11	None reported
5	35/F	DORV, ASD/VSD, CoA	Heart Failure, Cirrhosis, CKD, Anxiety, Depression	Aspirin, Metolazone, Nadolol, Spironolactone, Torsemide	Dulaglutide followed by semaglutide	2023	244, ongoing	43.6 − 3.0 kg, − 2.8%	+ 9	GI discomfort
6	31/F	HLHS	Hypertension, Depression	Aspirin, Metoprolol	Semaglutide	2023	219	52.5 − 11.4 kg, − 8.8%	− 8	GI discomfort
7	20/M	HLHS	OSA, Cirrhosis	Aspirin, Digoxin, Lisinopril	Liraglutide followed by semaglutide	2022	121	38.9 + 1.8 kg, + 1.5%	+ 5	None reported
8	37/M	TriA	Pacemaker, Anxiety	Lisinopril, Rivaroxaban, Tadalafil, Furosemide, Metoprolol, Spironolactone	Semaglutide	2023	339, ongoing	31.1 − 14.1 kg, − 15.5%	− 26	Headache, resolved

*ASD* atrial septal defect, *CKD* chronic kidney disease, *CoA* coarctation of the aorta, *DORV* double-outlet right ventricle, *HLHS* hypoplastic left heart syndrome, *MI* myocardial infarction, *OSA* obstructive sleep apnea, *PA/IVS* pulmonary atresia with intact ventricular septum, *PH* pulmonary hypertension, *TriA* tricuspid atresia, *VSD* ventricular septal defect

Five patients (62.5%) lost weight, and the median weight loss in this group was 5.6% of initial body weight (range 2.1% to 15.5%). Compared to baseline, 6 patients (75%) had experienced a decrease in systolic blood pressure by a median of 9.5 mmHg at the last documented visit. Side effects were reported in 4 patients (50%); 3 of these were gastrointestinal (GI) discomfort, and the remaining patient reported headaches. There were no reported severe side effects requiring hospitalization, such as pancreatitis. Early drug termination occurred in 3 patients. One termination was due to drug supply shortage, and this patient plans to restart the medication (Patient 1). One patient was unable to restart the medication after a brief pause in treatment because of GI side effects (Patient 6). The final patient stopped due to not losing weight on treatment (Patient 7).

## Discussion

We observed a modest weight loss and improvement in blood pressure in a small group of patients with Fontan circulation treated with GLP-1 agonists, the first report of their use in this population. It is established that these medicines can decrease weight, lower blood pressure, and may reduce endovascular inflammation and improve ventricular function in the general population [3]. These medications are thought to work through promoting insulin release, inhibiting glucagon secretion, and slowing gastric emptying even in the absence of diabetes [4]. Patients with Fontan circulation have been reported to have increased insulin resistance even without diabetes, increased markers of inflammation, and higher prevalence of diastolic dysfunction [5, 6]. These negative effects of Fontan physiology may be more attenuated by using GLP-1 agonists. Thus, there may be a potential for greater symptomatic benefit in patients with Fontan circulation because of these proposed mechanisms.

We found a median weight loss of 5.6%, lower than a recent review of 14 randomized controlled trials showing weight loss between 6.1 and 17.4% [4]. However, we have a smaller sample size and more heterogeneous follow-up than these studies. More recently, GLP-1 agonists have been shown to play an important role in the care of adults with heart failure with preserved ejection fraction (HFpEF), inducing weight loss and improvement in both heart failure-related symptoms and markers of inflammation and congestion [7]. Like patients with HFpEF, patients with Fontan circulation often have symptomatic heart failure in the setting of normal ejection fraction secondary to diastolic dysfunction [5].

As reported in the general population, the most common side effects were GI, occurring in up to 30% of these patients [3]. We found a similar proportion of patients with these side effects in our study, and only one patient discontinued the medication because of the severity of these side effects. It is reassuring that no severe side effects, such as pancreatitis or severe gastroparesis, occurred in this group. There were no observed negative interactions with other medications these patients took.

This series provides preliminary data supporting the safety and potential efficacy for weight loss of GLP-1 agonists in certain patients with Fontan circulation and concurrent obesity, with or without diabetes mellitus. Given the small sample size and limited duration of observed therapy, future longitudinal studies are required to validate these results and to understand the impact on heart failure in this population. Nevertheless, these observations provide optimism that these medications could be used as a tool to improve the cardiovascular and cardiometabolic health of patients with Fontan circulation.

## Data Availability

No datasets were generated or analysed during the current study.
